# A New Algorithm to Optimize Maximal Information Coefficient

**DOI:** 10.1371/journal.pone.0157567

**Published:** 2016-06-22

**Authors:** Yuan Chen, Ying Zeng, Feng Luo, Zheming Yuan

**Affiliations:** 1 Hunan Provincial Key Laboratory for Biology and Control of Plant Diseases and Insect Pests, Hunan Agricultural University, Changsha, China; 2 Orient Science &Technology College of Hunan Agricultural University, Changsha, China; 3 College of Plant Protection, Hunan Agricultural University, Changsha, China; 4 School of Computing, Clemson University, Clemson, South Carolina, United States of America; 5 Hunan Provincial Key Laboratory for Germplasm Innovation and Utilization of Crop, Hunan Agricultural University, Changsha, China; Indiana University Bloomington, UNITED STATES

## Abstract

The maximal information coefficient (MIC) captures dependences between paired variables, including both functional and non-functional relationships. In this paper, we develop a new method, ChiMIC, to calculate the MIC values. The ChiMIC algorithm uses the chi-square test to terminate grid optimization and then removes the restriction of maximal grid size limitation of original ApproxMaxMI algorithm. Computational experiments show that ChiMIC algorithm can maintain same MIC values for noiseless functional relationships, but gives much smaller MIC values for independent variables. For noise functional relationship, the ChiMIC algorithm can reach the optimal partition much faster. Furthermore, the MCN values based on MIC calculated by ChiMIC can capture the complexity of functional relationships in a better way, and the statistical powers of MIC calculated by ChiMIC are higher than those calculated by ApproxMaxMI. Moreover, the computational costs of ChiMIC are much less than those of ApproxMaxMI. We apply the MIC values tofeature selection and obtain better classification accuracy using features selected by the MIC values from ChiMIC.

## Introduction

Identifying relationships between variables is an important scientific task in exploratory data mining [1]. Many measures have been developed, such as Pearson correlation coefficient [2], Spearman rank correlation coefficient, Kendall coefficient of concordance [3], mutual information estimators [4],[5],[6], distance correlation (dCor) [7], and correlation along a generating curve [8]. Recently, Reshef *et al*. [9] proposed a novel maximal information coefficient (MIC) measure to capture dependences between paired variables, including both functional and non-functional relationships [10],[11]. The MIC value has been applied successfully to many problems [12–20]. The MIC of a pair of data series *x* and *y* is defined as follow:

$$MIC\left(x,y\right)=\text{max}\{I\left(x,y\right)/\log_{2}\;\text{min}\left\{n_{x},n_{y}\right\}\}$$ (1)

where *I*(*x*, *y*) is the mutual information between data *x* and *y*. The *n*_*x*_, *n*_*y*_ are the number of bins into which *x* and *y* are partitioned, respectively. Reshef *et al*. [9] developed a dynamic program algorithm, ApproxMaxMI, to calculate the MIC. As the bin sizes affect the value of mutual information, determining the appropriate value of *n*_*x*_ and *n*_*y*_ is important. In ApproxMaxMI algorithm, Reshef set the *n*_*x*_×*n*_*y*_˂ *B*(*n*), where *B*(*n*) = *n*^0.6^ is the maximal grid size restriction and *n* is the sample size. The generality of MIC is closely related to *B*(*n*). Setting *B*(*n*) too low will result in searching only for simple patterns and weakening the generality, while setting *B*(*n*) too high, will result in nontrivial MIC score for independent paired variables under finite samples. For example, the MIC value of two independent variables with 400 sample points calculated by ApproxMaxMI is 0.15±0.017 (*B*(*n*) = *n*^0.6^, 500 replicates). Furthermore, the computation cost of ApproxMaxMI becomes expensive when the sample size *n* is large, which makes it difficult to calculate the MIC for big data.

Many efforts have been committed to improve the approximation algorithm for MIC by either optimizing the MIC value or reducing the computing time. Tang *et al*. [21] have proposed a cross-platform tool for the rapid computation of the MIC based on parallel computing methods. Wang *et al*. [22] have used quadratic optimization to calculate the MIC. Albanese *et al*. [23] re-implemented the ApproxMaxMI using C.[9]. Zhang *et al*. [10] have applied the simulated annealing and genetic algorithm, SGMIC, to optimize the MIC. Although SGMIC can achieve better MIC values, it is much more time-consuming.

In this paper, we develop a new algorithm to improve the MIC values as well as reduce the computational cost. Our new algorithm, ChiMIC, uses the chi-square test to determinate optimal bin size for the calculating of MIC value. Experiments on simulated data with different relationships (*e*.*g*. statistically independent, noiseless functional and noisy functional relationships) and real data show that our method can optimize the MIC value and significantly reduce the computation time.

## Results

### Comparison of MIC values for independent and noiseless functional relationships

**Fig 1:**
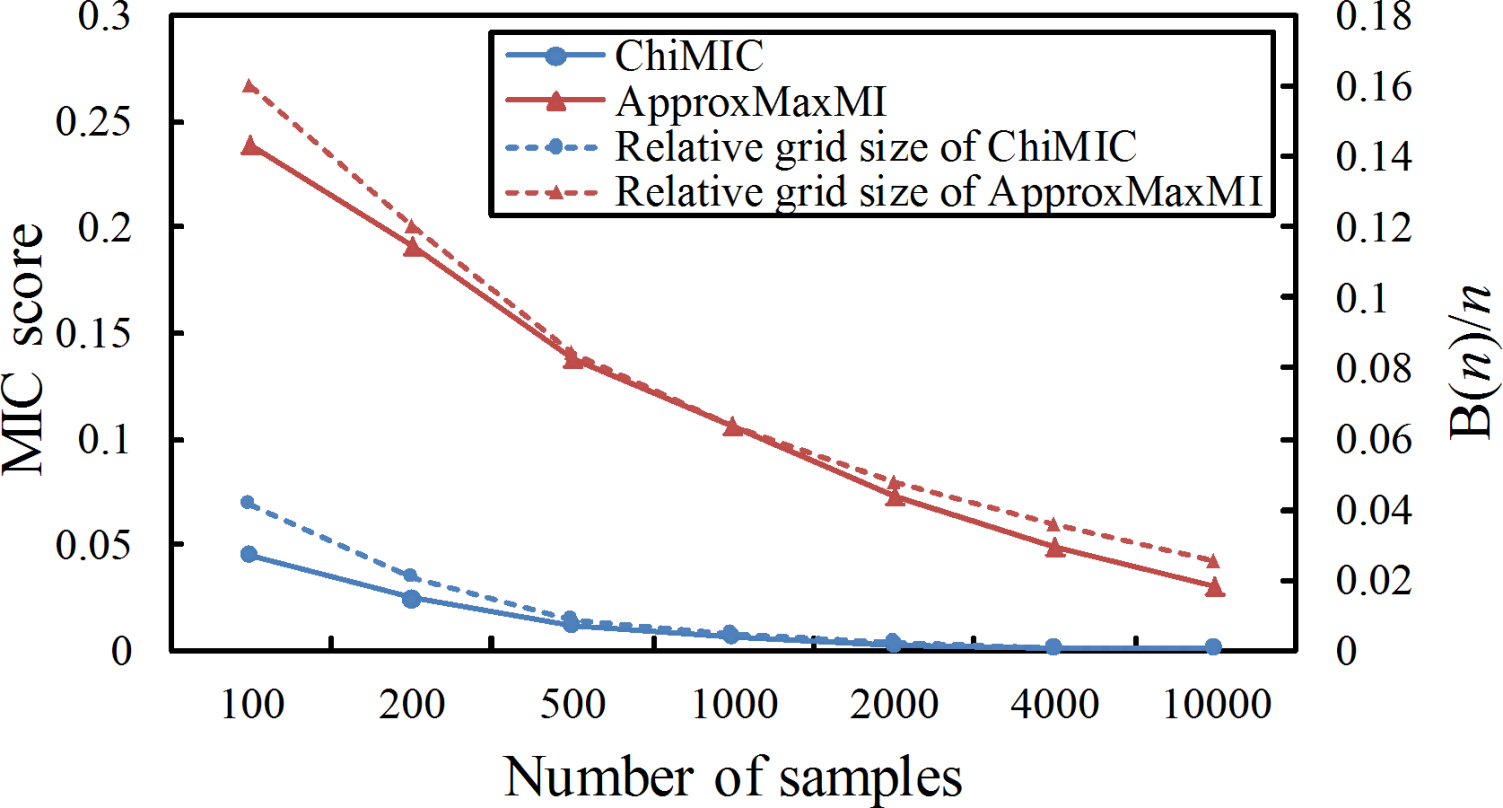
MIC values calculated by ApproxMaxMI and ChiMIC for random data with different sample sizes. The scores are reported as means over 500 replicates.

Ideally, the MIC values should be close to 0 for two independent variables. The MIC values for two independent variables calculated by ApproxMaxMI depend on the ratio between B(*n*) and *n* [24]. The MIC value for two independent variables is close to 0 only when *n* approaches infinity, namely, the B(*n*)/*n* trends to 0. Although, the MIC values calculated by ChiMIC also depend on *B*(*n*)/*n*, the ChiMIC algorithm gives much smaller MIC values for independent variables and converges to zero faster as shown in Fig 1. Specifically, for small sample sizes, the MIC values calculated by ChiMIC are much smaller. For example, when sample size is 100, MIC value calculated by ChiMIC is 0.06, while MIC value calculated by ApproxMaxMI is 0.24.

Meanwhile, the MIC values of two variables with noiseless functional relationships should be close to 1. We calculate the MIC values between variables with 21 different noiseless functional relationships (S1 Fig) using ChiMIC algorithm. All MIC values are equal to 1 as shown in S1 Table, which indicates that the ChiMIC algorithm maintains the generality of MIC.

### Comparison of grid partition for noisy relationships

**Fig 2:**
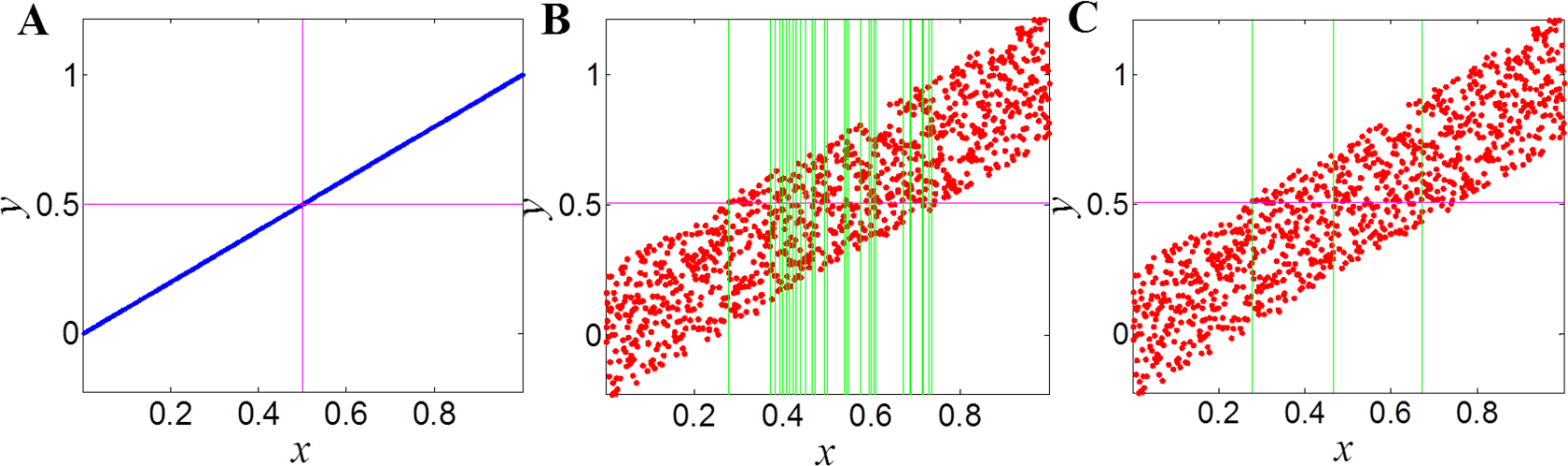
Grid partition of ApproxMaxMI and ChiMIC for linear function. 1000 data points simulated for functional relationships of the form *y* = *x*+*η*. where *η* is noise drawn uniformly from (−0.25, 0.25). A: Grid partition for noiseless linear function. B: Grid partition based on ApproxMaxMI for noisy linear function. C: Grid partition based on ChiMIC for noisy linear function.

**Fig 3:**
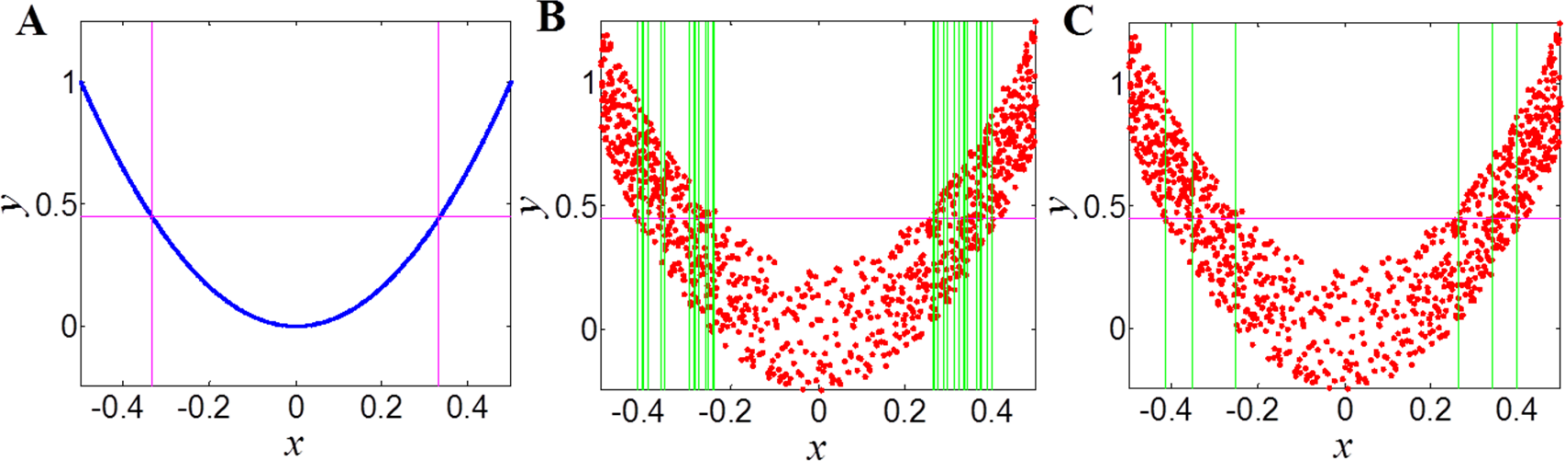
Grid partition of ApproxMaxMI and ChiMIC for parabolic function. 1000 data points simulated for functional relationships of the form *y* = 4*x*^*2*^+*η*. where *η* is noise drawn uniformly from (−0.25, 0.25). A: Grid partition for noiseless parabolic function. B: Grid partition based on ApproxMaxMI for noisy parabolic function. C: Grid partition based on ChiMIC for noisy parabolic function.

**Fig 4:**
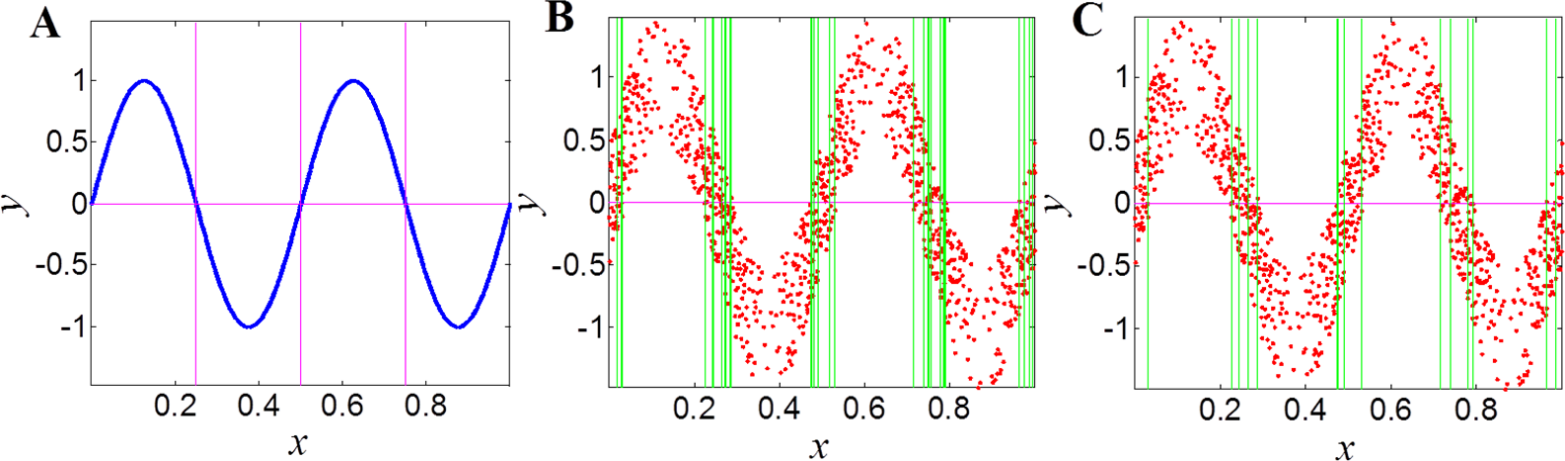
Grid partition of ApproxMaxMI and ChiMIC for sinusoidal function. 1000 data points simulated for functional relationships of the form *y* = sin(4π*x*)+*η*. where *η* is noise drawn uniformly from (−0.25, 0.25). A: Grid partition for noiseless sinusoidal function. B: Grid partition based on ApproxMaxMI for noisy sinusoidal function. C: Grid partition based on ChiMIC for noisy sinusoidal function.

For noise relationships, the ChiMIC algorithm terminates the grid partition search much earlier than ApproxMaxMI. As shown in Fig 2, both ChiMIC and ApproxMaxMI capture a noiseless linear relationship with a 2×2 grid (Fig 2A). When adding noise, the ApproxMaxMI partitions the noisy linear relationship with a 2×32 grid (Fig 2B). Meanwhile, ChiMIC partitions the noisy linear relationship with just a 2×4 grid (Fig 2C). We also compare the grid partition for parabolic function (Fig 3) and sinusoidal function (Fig 4). As shown in Figs 3 and 4, the ChiMIC algorithm partitions the noisy functional relationship into much fewer numbers of grids. The fewer numbers the grid are partitioned, the smaller the MIC values are calculated. The MIC values calculated by ChiMIC algorithm are always smaller than those calculated by ApproxMaxMI. We calculate the MIC values for 21 functional relationships with different noise levels (S2 Table). The higher the noise level, the smaller the MIC values calculated by ChiMIC comparing to those calculated by ApproxMaxMI.

### Comparison of minimum cell number (MCN) estimation

In maximal information-based nonparametric exploration statistics, the minimum cell number (MCN) is the number of grid cells needed to calculate MIC values. It is defined as follows [24]:

$$\text{MCN}(D,\varepsilon )=\underset{xy<B}{\min\{log_{2}(xy):M{\left(D\right)}_{x,y}\geq (1-\varepsilon )MIC(D)\}}$$ (2)

where *D* is a finite set of ordered paired data. The parameter*ε* provides robustness for MCN. This measure captures the complexity of the association between two variables. A greater MCN measure indicates a more complex association.

**Fig 5:**
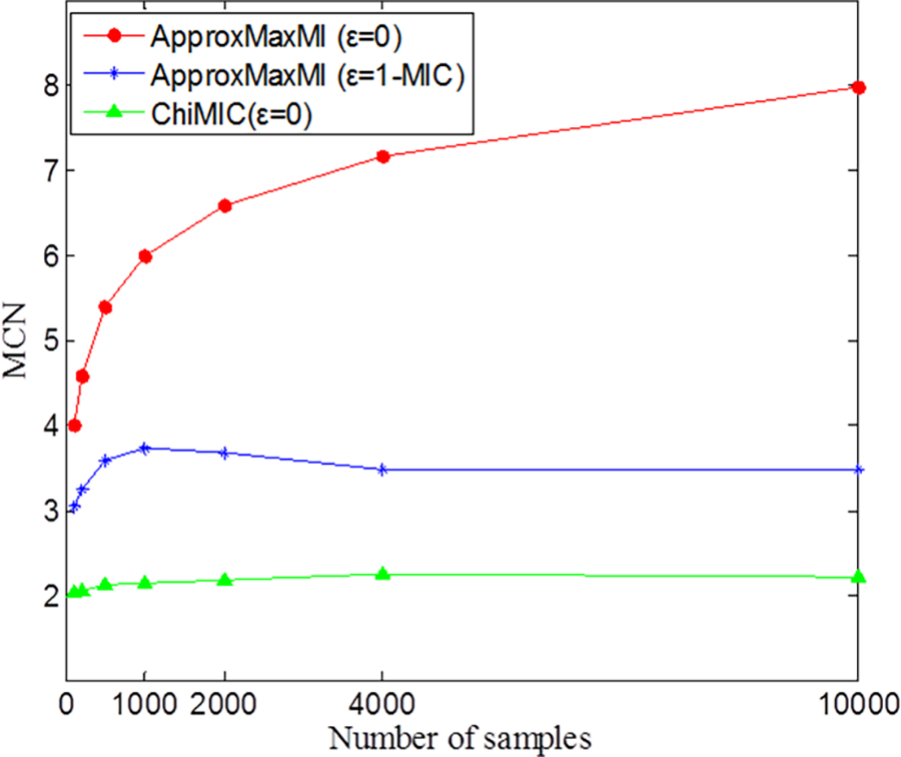
MCN values of independent variables for different sample sizes. The values are reported as means over 500 replicates.

For independent variables, MCN is equal to 2 and is unrelated to sample size. When *ε* is set to 0, the MCN measure based on MIC calculated by ApproxMaxMI increases steadily as the sample size grows (Fig 5). When *ε* is set to 1-MIC(D) as in Reshef [24], the MCN measures based on MIC calculated by ApproxMaxMI algorithm do not increase as sample size grows, but still maintain greater than 3. On the other hand, the MCN values based on MIC calculated by ChiMIC are always close to 2.

**Fig 6:**
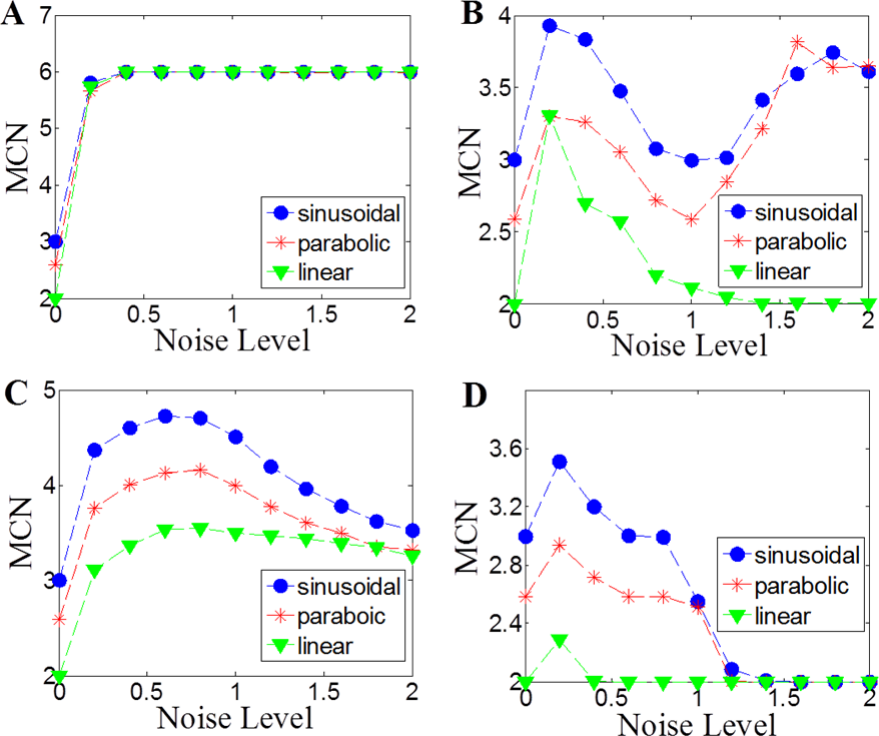
MCN values for linear, parabolic and sinusoidal functions at different noise levels. A MCN estimates with MIC (*ε* = 0), B MCN estimates with MIC (*ε* = 1-MIC), C MCN estimates for ChiMIC (*ε* = 0), D MCN estimates for ChiMIC (*ε* = 1-MIC). MCN estimates were computed for n = 1000 data points over 500 replicates. Each relationship listed, is the same as in Figs 2–4. Where *y* = *f*(*x*)+*η*, *η* was uniform noise of amplitude equal to the range of *f*(*x*) times one of these 12 relative amplitudes:0, 0.2, 0.4, 0.6, 0.8, 1.0, 1.2,1.4, 1.6, 1.8, 2.0.

For noiseless linear, parabolic and sinusoidal functions, the MCN values are 2, 2.58 and 3, respectively, for MIC calculated by either ApproxMaxMI or ChiMIC (Fig 6). As MCN values increase as the complexity of functional relationships increases, the MCN values should increase when weak noise is added. However, when the level of noise blurs the real functional relationship, the MCN values should decrease and converge towards 2. Thus, the MCN values should follow a parabolic graph as the noise level increases. We examine the MCN values when different levels of noise are added to linear, parabolic and sinusoidal functions. When *ε* is set to 0 and noise level is greater than or equal to 0.4, the MCN values based on MIC calculated by ApproxMaxMI are always equal to 6 for all three functions (Fig 6A). Thus, MCN can no longer capture the complexity of functional relationships in this case. When *ε* is set to 1-MIC(D), only the MCN values based on MIC calculated by ApproxMaxMI for linear function follow the parabolic form. The MCN values based on MIC calculated by ApproxMaxMI for parabolic and sinusoidal functions do not follow the parabolic form, and cannot reflect the complexity of functional relationships under some noise levels (Fig 6B). When *ε* is set to 0, MCN values based on MIC calculated by ChiMIC for all three functions follow the parabolic form (Fig 6C). However, the MCN values do not converge to 2. When *ε* is set to 1-MIC(D), MCN values based on MIC calculated by ChiMIC for all three functions not only follow the parabolic form, but also converge to 2 when noise reaches in certain level. These results imply that MCN values based on MIC calculated by ChiMIC can capture the complexity of functional relationships in a better way.

### Comparison of statistical power

The power of a statistical test is an important concept in hypotheses testing [25]. The empirical statistical power is the proportion of tests that correctly reject the null hypothesis. dCor is considered to be a dependency measures with high statistical power [25],[26],[27], so we compare the statistical powers of MIC calculated by ApproxMaxMI and ChiMIC with those of dCor.

**Fig 7:**
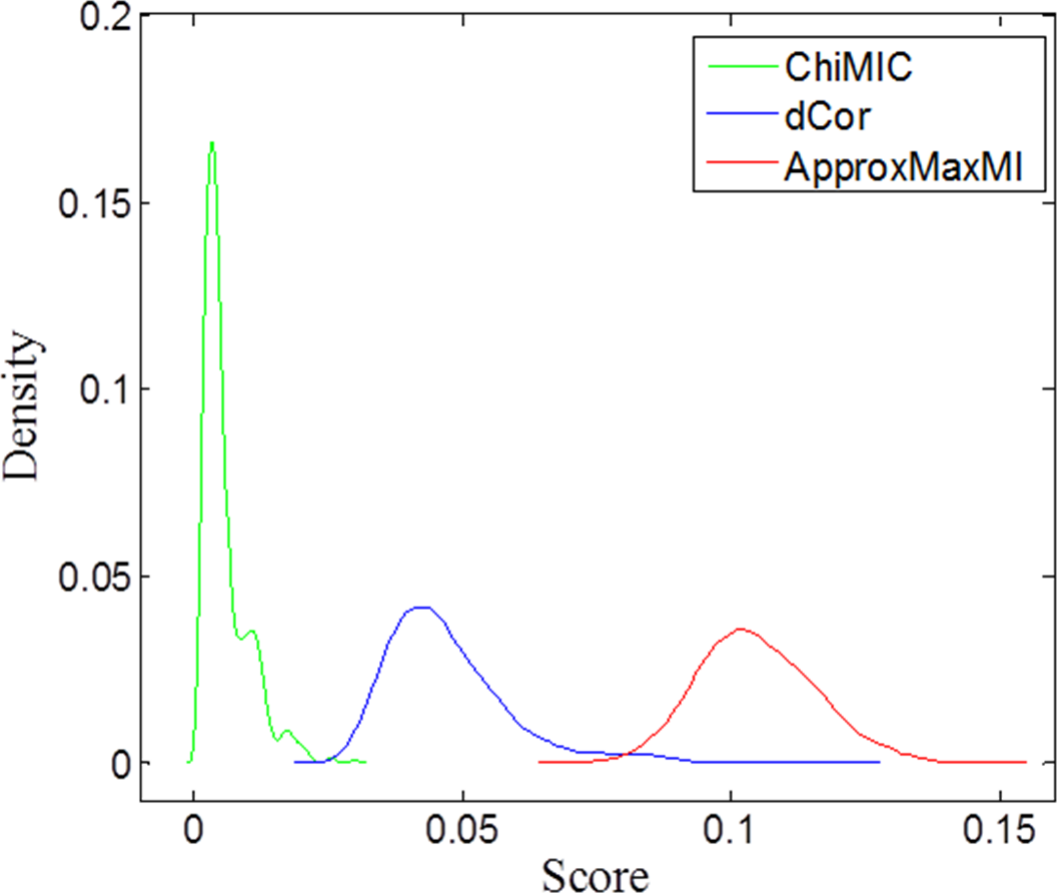
Density distribution of ApproxMaxMI, ChiMIC and dCor scores for two independent variables. ApproxMaxMI, ChiMIC and dCor estimates were computed for sample size *n* = 400 over 1000 replicates.

For the null hypothesis of statistical independency, a larger average value and standard deviation for the dependency measure indicate lower statistical power. Therefore, for two independent variables, a good dependency measure should have small standard deviation and zero average value, although small average value and standard deviation do not directly mean high statistical power. Fig 7 illustrates the density distribution of MIC values calculated by ApproxMaxMI and ChiMIC, as well as the dCor scores for the null hypothesis. Obviously, MIC values calculated by ChiMIC have a smaller average value and standard deviation. Therefore, they potentially have higher statistical power.

**Fig 8:**
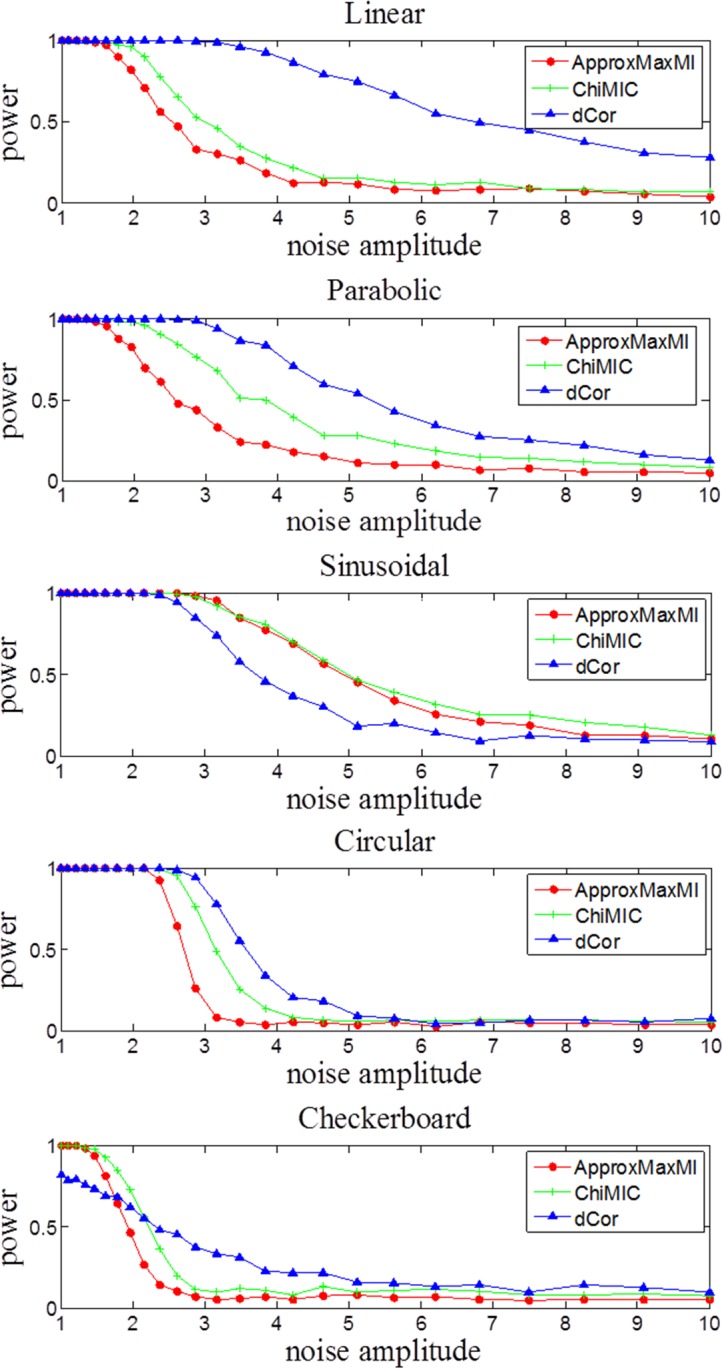
Statistical power of MIC from ApproxMaxMI, ChiMIC and dCor with different levels of noise, for five kinds of functional relationships. The statistical power was estimated via 500 simulations, with sample size *n* = 400.

However, the statistical power of dependency measures may depend on different factors, such as pattern types, noise levels and sample sizes. We examine the statistical power of dCor and MIC calculated by ApproxMaxMI and ChiMIC for five different functions with different noise levels. As shown in Fig 8, the statistical powers of MIC calculated by ChiMIC are higher than those of MIC calculated by ApproxMaxMI for all five functional relationships. For linear, parabolic and circular functional relationships, dCor has higher statistical power than those of MIC calculated by ApproxMaxMI and ChiMIC. For sinusoidal function, statistical power of MIC calculated by ApproxMaxMI and ChiMIC are both higher than that of dCor. For checkerboard function, MIC calculated by ApproxMaxMI and ChiMIC outperform dCor at low noise levels, while dCor outperforms at high noise levels.

### Comparison of computational cost of ApproxMaxMI and ChiMIC

The computational cost of MIC increases as the sample size *n* increases. With more and more big data available, the computational cost becomes critical for MIC calculating algorithm. We compare the computational time of ChiMIC and ApproxMaxMI using different sizes of independent variable pairs. As shown in Table 1, the run times of ChiMIC algorithm are significantly less than those of ApproxMaxMI algorithm. This is because ChiMIC algorithm uses the chi-square test to terminate grid optimization earlier, while ApproxMaxMI algorithm always tends to search to the maximal grid size B(*n*). When sample size is 100, ChiMIC algorithm is about 30% faster than ApproxMaxMI algorithm. As samples sizes increase, the advantage of ChiMIC algorithm becomes even more evident. When the sample size is 20000, ChiMIC algorithm runs nearly five times faster than ApproxMaxMI algorithm does. For the parabolic function with noise_LEVEL is 0.4, ChiMIC algorithm also has a faster convergence speed. Thus, ChiMIC algorithm will be a better method for calculating MIC values of big data.

**Table 1:** Elapsed time for calculating MICs for different sample sizes.

	Number of samples	100	1000	2000	4000	10000	20000
Independent variable pair	ChiMIC (second)	0.0046±5e-04	0.1520±0.03	0.537±0.11	2.166±0.59	12.93±4.1	53.55±11
	ApproxMaxMI(second)	0.0061±6e-04	0.5229±0.02	2.188±0.24	8.502±0.12	60.02±0.8	284.38±11
Parabolic function (noise_LEVEL = 0.4)	ChiMIC (second)	0.0021±3e-04	0.1325±0.02	0.5946±0.12	2.754±0.45	21.41±3.5	99.70±10
	ApproxMaxMI(second)	0.0021±5e-04	0.1407±0.03	0.7360±0.15	3.523±0.54	26.65±2.1	122.8±12

The corresponding time was represented as the average value ± standard deviation over 100 time replicated runs on a Windows 7 32-bit operating system (RAM: 3.00GB, CPU: 2.80 GHz).

### Application of MIC values to selecting features for cancer classification

The dependency measures can be used to select features. We compare the MIC values calculated by ApproxMaxMI and ChiMIC in feature selection. We test feature selection on four cancer classification data sets: two microarray datasets (Prostate1 and Prostate2) and two image datasets (WDBC and WPBC). We partition each dataset into a training set and a test set randomly (see details in the Method section). The training set is used for feature selection and classifier construction, and the test set is used for model validation. The retained features and independent test accuracies for the four datasets are shown in Table 2. For the Prostate1 dataset, although the methods based on MIC values from ApproxMaxMI and ChiMIC both select four features, features selected by MIC values from ChiMIC give much higher test accuracy (93.33% vs. 86.67%). For the other three data sets, MIC values from ChiMIC select fewer features than those selected by MIC values from ApproxMaxMI. All classifiers based on features selected by MIC values from ChiMIC obtain higher test accuracy. Especially for the Prostate2 dataset, the test accuracy of classifier based on features selected by MIC values from ChiMIC (80.77) is 11.54% higher than that of classifier based on features selected by MIC values from ApproxMaxMI (69.23%). This result suggests that the features selected by MIC values from ChiMIC are more effective.

**Table 2:** Retained features and independent test accuracy based on MIC and ChiMIC.

Datasets	Feature selection method	Selected features	Feature size	Test accuracy (%)
Prostate1	ApproxMaxMI	TRGC2, SERPINF1, NELL2, SPON1	4	86.67
	ChiMIC	TRGC2, HPN, NELL2, LMO3	4	**93.33**
Prostate2	ApproxMaxMI	SUCO, SMARCD3, XRCC5, PXDC1, CCL2, MAGEA11, MFAP3L, ABCC3, HLA-DPB1	9	69.23
	ChiMIC	SMARCD3, CCND1, ESR1, SSH2, PISD, BRD2, DIS3, RASA3	**8**	**80.77**
WDBC	ApproxMaxMI	Concave points SE, Worst concave points, Perimeter SE, Mean fractal dimension, Mean perimeter, Mean texture, Worst Radius, Compactness SE, Worst compactness, Radius SE, Mean area	11	97.06
	ChiMIC	Concave points SE, Worst concave points, Perimeter SE, Mean fractal dimension, Worst Radius, Worst compactness, Radius SE, Worst fractal dimension	**8**	**97.65**
WPBC	ApproxMaxMI	Mean Radius, Worst compactness, Mean perimeter, Worst concave points, Worst fractal dimension, Symmetry SE, Mean smoothness, Mean compactness, Mean concavity	9	72.88
	ChiMIC	Mean Radius, Symmetry SE, Worst concave points, Mean concave points, Worst compactness, Fractal dimension SE	**6**	**76.27**

## Methods

### ChiMIC algorithm: determining optimal grid using chi-square test

**Fig 9:**
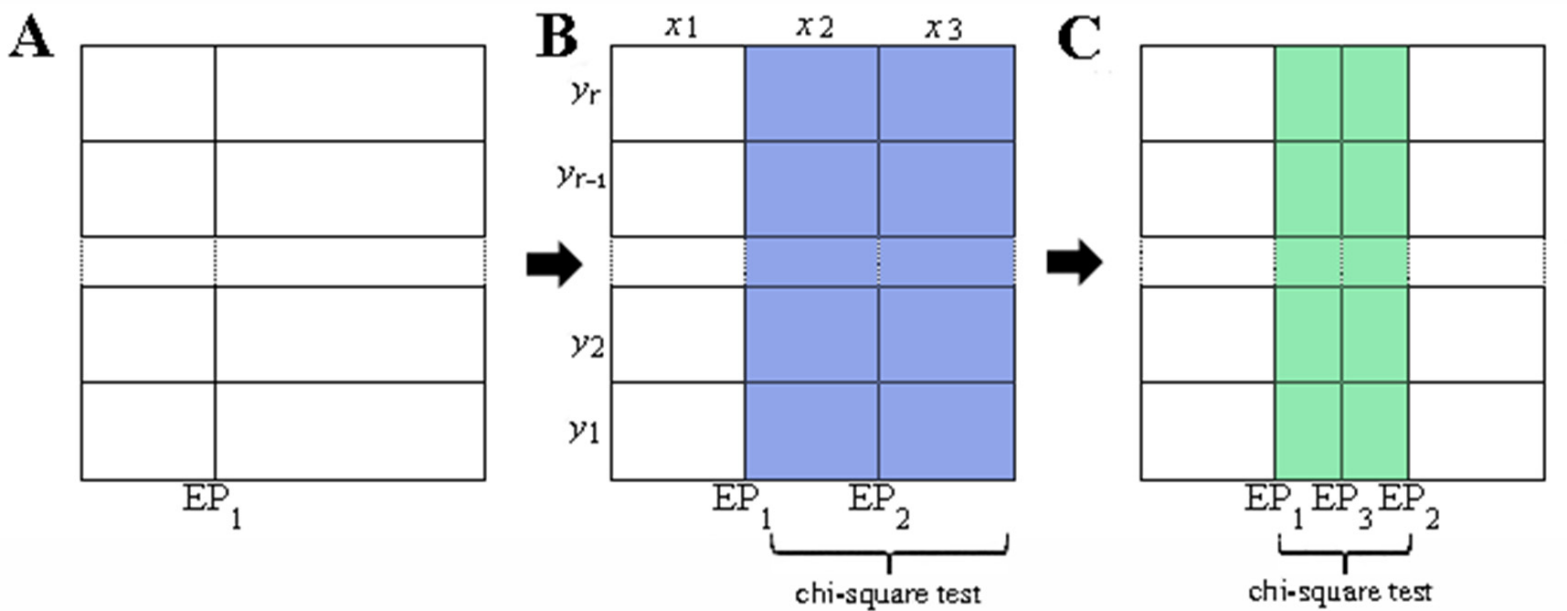
Illustration of x-axis partition of ChiMIC. Colored *r*×2 contingency tables are used for chi-square test.

Same as ApproxMaxMI [9] algorithm, the ChiMIC algorithm partitions a data set of ordered pairs through *x*-axis and *y*-axis. Similar to ApproxMaxMI [9] algorithm, the ChiMIC algorithm tries to find optimal partition of *x*-axis given an equipartition of *r* bins on *y*-axis. For example, in Fig 9A, the first optimal endpoint EP_1_ divides *x*-axis into two bins. Then, we will use a chi-square test to determine whether the next endpoint is useful. If the *p*-value of chi-square test is lower than a given threshold, the endpoint is useful and the ChiMIC algorithm continues searching for next optimal endpoint. On the other hand, if the *p*-value of chi-square test is greater than the given threshold, the endpoint is not useful and the process of partition *x*-axis is terminated.

The chi-square statistic is defined in Eq (3):

$$\chi_{{}_{EP_{m}}}^{2}=\sum_{i=1}^{r}\sum_{j=k}^{k+1}\frac{{\left(f_{ij}-n_{i}T_{j}/N\right)}^{2}}{n_{i}T_{j}/N}$$ (3)

where the *m*^*th*^(*m>*1) new endpoint EP_*m*_ divides *x*-axis into the *k*^*th*^ and *(k+1)*^*th*^ bin. *i* (*i* = 1,2,…,*r*) denotes the *i*^th^ bin of *y*-axis, *j*(*j* = *k*, *k+*1) denotes the *j*^th^ bin of *x*-axis. *f*_*ij*_ denotes the number of sample points falling into the cell in the *i*^th^ row and *j*^th^ column. *n*_*i*_ denotes the number of sample points falling in the *i*^th^ row, *T*_*j*_ denotes the number of sample points falling in the *j*^th^ column, *N* denotes the total number of sample points falling in the *k*^*th*^ and *(k+1)*^*th*^ columns. For example, in Fig 9B, the second optimal endpoint EP_2_ is selected by dynamic programming. The sample points distributed in the blue area (the *x*_2_^th^ column and *x*_3_^th^column in Fig 9B) are used to perform a chi-square test on an *r*×2 contingency table. If EP_2_ is useful, then ChiMIC continues searching for the next optimal endpoint. If the next one is EP_3_, we use the sample points distributed in the green area to perform a chi-square test on an *r*×2 contingency table (Fig 9C). If *p*-value is greater than the given threshold, the optimizing process is terminated. For the chi-square test, the minimum expected count in each group is five [28]. The *p*-value of chi-square test for a 2×2 table with data counts {0, 5; 5, 0} is 0.0114. So we choose 0.01 as the threshold. The Chi-square value needs correction for continuity when *r* = 2 [29].

### Adding noise to functional relationship

The noise to functional relationships in S1 Fig, Fig 2, Fig 3, Fig 4 and Fig 6 are defined as follows:

Y=f(X)+(RAND(N,1)–0.5)×noise_LEVEL×RANGE (4)

where *f* is a functional relationship, and RANGE represents the range of *f*(*X*). noise_LEVEL is the relative amplitudes: 0, 0.2, 0.4, 0.6, 0.8, 1.0, 1.2,1.4, 1.6, 1.8, 2.0. RAND(*N*, 1) is used to generate *N* random numbers in [0, 1].

The noise functional relationships in Fig 8 are defined in Table 3. Five hundred trial datasets are generated for each of these relationships at each of twenty-five different noise amplitudes (*a*) distributed logarithmically between 1 and 10. For each dataset, statistics are computed on the “true” data {*X*_*i*_, *Y*_*i*_}(*i* = 1,…,400) as well as on “null” data, for which the indices *i* on the *y* values are randomly permuted. The power of each statistic is defined as the fraction of true datasets yielding a statistic value greater than 95% of the values yielded by the corresponding null datasets. *ξ* and *η* are random numbers drawn from the normal distribution *N*(0,1). *θ* is a random number drawn uniformly from the interval [−π, π). (*X*_0_,*Y*_0_) is a pair of random numbers drawn uniformly from the solid squares of a 4×5 checkerboard, where each square has sides of length 1[26].

**Table 3:** The *X*, *Y* relationships simulated for the power calculations in Fig 8.

Relationship	*X*	*Y*
Linear	*ξ*	2/3*X* +*aη*
Parabolic	*ξ*	*X*2 + *aη*
Sinusoidal	5/2 *θ*	2 cos(*X*) + *aη*
Circular	10 cos(*θ*) + *aξ*	10 sin(*θ*) + *aξ*
Checkerboard	10 *X*_0_ + *aξ*	10*Y*_0_ + *aη*

### Real datasets

We use four real datasets to validate the proposed approach. Prostate1 [30] and Prostate2 [31] are gene expression profile datasets. Wisconsin Diagnostic Breast Cancer (WDBC) and Wisconsin Prognostic Breast Cancer (WPBC) [32] are image data of tumor tissues obtained from UCI database, which can be download at http://archive.ics.uci.edu/ml/data. The number of features, positive and negative samples in both training set and test set are listed in Table 4.

**Table 4:** Datasets: Training set and test set are divided randomly at a ratio of 7:3.

Dataset	Data type	Feature number	Training set		Test set	
			positive samples	negative samples	positive samples	negative samples
prostate1	microarray	12600	35	37	15	15
prostate2	microarray	12625	35	27	15	11
WDBC	image feature	32	250	149	107	63
WPBC	image feature	34	106	33	45	14

### Feature selection

For each dataset, first, we calculate MIC values of a vector (*X*, *Y*) separately in training set, where *X* denotes the value of each feature, and *Y* denotes the phenotype of tumors. Then, we rank all the features in descending order of MIC values. Next, we sequentially introduce the ranked features (only top 200 features for datasets Prostate1 and Prostate2) and remove redundant features using 10-fold cross-validation based on support vector classification (SVC). Finally, we build a SVC prediction model based on retained features using training data and perform independent prediction on test data.

### Computational Methods

The ChiMIC (S1 File) and ApproxMaxMI algorithms are both implemented in Matlab. The parameters of ApproxMaxMI are set as *a* = 0.6, *c* = 5. dCor and statistical power are computed using Matlab scriptsdownloaded at http://www.sourceforge.net [26]. The SVC is performed using LIBSVM [33] described by Chang *et al*, and can be downloaded at http://www.csie.ntu.edu.tw/~cjlin/libsvm/index.html.

## Supporting Information

S1 Fig 21 Noiseless functions. (DOCX)

S1 File This is the Chi*MIC* Matlab code. (ZIP)

S1 Table 21 Functional relationships. (DOCX)

S2 Table MIC values of each functional relationship with noise. (DOCX)
