# Supplementary figures and images for: A New Algorithm to Optimize Maximal Information Coefficient
Source: PLoS One. 2016 Jun 22;11(6):e0157567. doi: 10.1371/journal.pone.0157567 (PMC4917098; doi:10.1371/journal.pone.0157567)

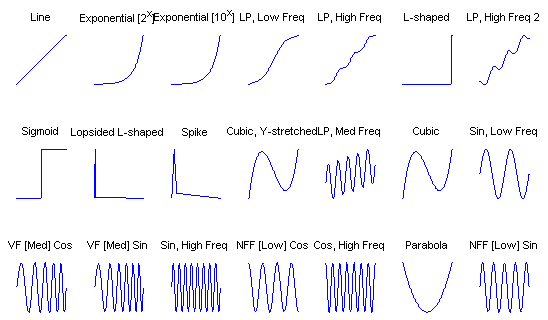


S1 Fig. 21 Noiseless functions22

Supplement: S1 Fig — (DOCX) [file pone.0157567.s001.docx]
