# Supplementary material for: A New Algorithm to Optimize Maximal Information Coefficient
Source: PLoS One. 2016 Jun 22;11(6):e0157567. doi: 10.1371/journal.pone.0157567 (PMC4917098; doi:10.1371/journal.pone.0157567)
Supplement: S1 Table — (DOCX) [file pone.0157567.s003.docx]

S1 Table 21 Functional relationships

| Description | X | *f*(X) | **ApproxMaxMI** | **ChiMIC** |
| --- | --- | --- | --- | --- |
| Line | [0,1] | *X* | 1.0 | 1.0 |
| Exponential [2*^X^* ] | [0,10] | 2*^X^* | 1.0 | 1.0 |
| Exponential [10*^X^* ] | [0,10] | 10*^X^* | 1.0 | 1.0 |
| LP, low frequency | [0,1] | $\frac{1}{5}\sin(4(2X-1))+\frac{11}{10}(2X-1)$ | 1.0 | 1.0 |
| LP, high frequency | [0,1] | $\frac{1}{10}\sin(10.6(2X-1))+\frac{11}{10}(2X-1)$ | 1.0 | 1.0 |
| L shaped | [0,1] | $\left\{\begin{matrix} X/99 \text{ if }X\leq0.99 \\ \\ \\ 99X-98 \text{if }X>0.99 \end{matrix}\right.$ | 1.0 | 1.0 |
| LP, high frequency 2 | [0,1] | $\frac{1}{5}\sin(10.6(2X-1))+\frac{11}{10}(2X-1)$ | 1.0 | 1.0 |
| Sigmoid | [0,1] | $\left\{\begin{matrix} 0 \text{ if }X<0.49 \\ \\ \\ 50\text{（}X-0.5\text{）}+0.5 \text{if 0.49}\leq X<0.49 \\ \\ \\ 1 \text{ if }X\geq0.51 \end{matrix}\right.$ | 1.0 | 1.0 |
| Lopsided L shaped | [0,1] | $\left\{\begin{matrix} 200X \text{ if }X<0.005 \\ \\ \\ -198X+1.99 \text{if 0.005}\leq X<0.01 \\ \\ \\ -\frac{X}{99}+ \frac{1}{99} \text{ if }X\geq0.01 \end{matrix}\right.$ | 1.0 | 1.0 |
| Spike | [0,1] | $\left\{\begin{matrix} 20X \text{if }X<0.05 \\ \\ \\ -18X+1.9 \text{if 0.05}\leq X<0.1 \\ \\ \\ -\frac{X}{9}+ \frac{1}{9} \text{ if }X\geq0.1 \end{matrix}\right.$ | 1.0 | 1.0 |
| Cubic, Y stretched | [-1.3,1.1] | 41(4*X*^3^+*X*^2^-4X) | 1.0 | 1.0 |
| LP, medium frequency | [0,1] | sin(10π*X*)+*X* | 1.0 | 1.0 |
| Cubic | [-1.3,1.1] | 4*X*^3^+*X*^2^-4X | 1.0 | 1.0 |
| Sin, low frequency | [0,1] | sin(4π*X*) | 1.0 | 1.0 |
| VF [med] cos | [0,1] | cos(5π*X*(1+*X*)) | 1.0 | 1.0 |
| VF [med] sin | [0,1] | cos(6π*X*(1+*X*)) | 1.0 | 1.0 |
| Sin, high frequency | [0,1] | sin(16π*X*) | 1.0 | 1.0 |
| NFF [low] cos | [0,1] | cos(7π*X*) | 1.0 | 1.0 |
| Cos, high frequency | [0,1] | cos(14π*X*) | 1.0 | 1.0 |
| Parabola | [-0.5,0.5] | 4*X*^2^ | 1.0 | 1.0 |
| NFF [low] sin | [0,1] | sin(9π*X*) | 1.0 | 1.0 |
