# Supplementary material for: A New Algorithm to Optimize Maximal Information Coefficient
Source: PLoS One. 2016 Jun 22;11(6):e0157567. doi: 10.1371/journal.pone.0157567 (PMC4917098; doi:10.1371/journal.pone.0157567)
Supplement: S2 Table — (DOCX) [file pone.0157567.s004.docx]

S2 Table MIC values of each functional relationship with noise

| Noise Level | Line | | Exponential [2^X^ ] | | Exponential [10^X^ ] | | LP,low frequency | |
| --- | --- | --- | --- | --- | --- | --- | --- | --- |
|  | ApproxMaxMI | ChiMIC | ApproxMaxMI | ChiMIC | ApproxMaxMI | ChiMIC | ApproxMaxMI | ChiMIC |
| 0 | 1 | 1 | 1 | 1 | 1 | 1 | 1 | 1 |
| 0.05 | 0.97 | 0.96 | 0.96 | 0.96 | 0.97 | 0.97 | 0.97 | 0.97 |
| 0.1 | 0.95 | 0.93 | 0.95 | 0.93 | 0.94 | 0.93 | 0.95 | 0.94 |
| 0.2 | 0.92 | 0.89 | 0.89 | 0.84 | 0.91 | 0.87 | 0.93 | 0.88 |
| 0.3 | 0.87 | 0.81 | 0.85 | 0.78 | 0.85 | 0.79 | 0.89 | 0.81 |
| 0.4 | 0.81 | 0.74 | 0.77 | 0.70 | 0.78 | 0.72 | 0.84 | 0.76 |
| 0.5 | 0.71 | 0.63 | 0.74 | 0.64 | 0.75 | 0.67 | 0.76 | 0.68 |
| 0.6 | 0.66 | 0.57 | 0.69 | 0.59 | 0.67 | 0.60 | 0.73 | 0.66 |
| 0.7 | 0.61 | 0.52 | 0.62 | 0.53 | 0.60 | 0.52 | 0.67 | 0.58 |
| 0.8 | 0.53 | 0.45 | 0.54 | 0.45 | 0.55 | 0.45 | 0.57 | 0.50 |
| 0.9 | 0.49 | 0.40 | 0.50 | 0.41 | 0.52 | 0.44 | 0.54 | 0.46 |
| 1 | 0.43 | 0.35 | 0.49 | 0.39 | 0.45 | 0.36 | 0.45 | 0.39 |
| 1.15 | 0.41 | 0.32 | 0.37 | 0.29 | 0.45 | 0.37 | 0.46 | 0.37 |
| 1.3 | 0.36 | 0.28 | 0.34 | 0.25 | 0.37 | 0.29 | 0.41 | 0.33 |
| 1.45 | 0.31 | 0.25 | 0.35 | 0.27 | 0.33 | 0.26 | 0.35 | 0.28 |
| 1.6 | 0.30 | 0.23 | 0.32 | 0.24 | 0.31 | 0.23 | 0.33 | 0.25 |
| 1.8 | 0.31 | 0.22 | 0.29 | 0.21 | 0.30 | 0.20 | 0.31 | 0.24 |
| 2.1 | 0.24 | 0.16 | 0.26 | 0.17 | 0.25 | 0.17 | 0.31 | 0.21 |
| 2.5 | 0.25 | 0.16 | 0.26 | 0.16 | 0.25 | 0.17 | 0.24 | 0.17 |
| 3.1 | 0.21 | 0.13 | 0.19 | 0.11 | 0.22 | 0.13 | 0.21 | 0.13 |
| 4.5 | 0.19 | 0.09 | 0.18 | 0.09 | 0.16 | 0.08 | 0.21 | 0.10 |
| 6 | 0.14 | 0.05 | 0.13 | 0.01 | 0.16 | 0.06 | 0.19 | 0.07 |
| 8 | 0.12 | 0.04 | 0.13 | 0.04 | 0.12 | 0.04 | 0.14 | 0.05 |
| 10 | 0.12 | 0.04 | 0.14 | 0.06 | 0.12 | 0.04 | 0.10 | 0.03 |
| 20 | 0.11 | 0.01 | 0.12 | 0.03 | 0.12 | 0.00 | 0.13 | 0.03 |

| Noise Level | LP, high frequency | | L shaped | | LP, high frequency 2 | | Sigmoid | |
| --- | --- | --- | --- | --- | --- | --- | --- | --- |
|  | ApproxMaxMI | ChiMIC | ApproxMaxMI | ChiMIC | ApproxMaxMI | ChiMIC | ApproxMaxMI | ChiMIC |
| 0 | 1 | 1 | 1 | 1 | 1 | 1 | 1 | 1 |
| 0.05 | 0.97 | 0.96 | 0.98 | 0.92 | 0.98 | 0.97 | 0.99 | 0.99 |
| 0.1 | 0.97 | 0.94 | 0.96 | 0.85 | 0.95 | 0.94 | 0.97 | 0.97 |
| 0.2 | 0.90 | 0.89 | 0.93 | 0.76 | 0.93 | 0.91 | 0.95 | 0.93 |
| 0.3 | 0.86 | 0.80 | 0.85 | 0.71 | 0.89 | 0.82 | 0.92 | 0.90 |
| 0.4 | 0.82 | 0.74 | 0.80 | 0.63 | 0.83 | 0.77 | 0.91 | 0.86 |
| 0.5 | 0.72 | 0.65 | 0.78 | 0.58 | 0.75 | 0.68 | 0.88 | 0.82 |
| 0.6 | 0.65 | 0.56 | 0.75 | 0.51 | 0.68 | 0.59 | 0.81 | 0.76 |
| 0.7 | 0.60 | 0.53 | 0.66 | 0.50 | 0.61 | 0.53 | 0.83 | 0.75 |
| 0.8 | 0.53 | 0.46 | 0.61 | 0.41 | 0.60 | 0.51 | 0.77 | 0.71 |
| 0.9 | 0.49 | 0.42 | 0.52 | 0.37 | 0.54 | 0.44 | 0.77 | 0.70 |
| 1 | 0.47 | 0.39 | 0.47 | 0.38 | 0.48 | 0.40 | 0.68 | 0.60 |
| 1.15 | 0.44 | 0.34 | 0.42 | 0.33 | 0.45 | 0.37 | 0.57 | 0.49 |
| 1.3 | 0.40 | 0.31 | 0.36 | 0.26 | 0.39 | 0.30 | 0.55 | 0.47 |
| 1.45 | 0.33 | 0.25 | 0.35 | 0.26 | 0.39 | 0.30 | 0.47 | 0.39 |
| 1.6 | 0.36 | 0.27 | 0.33 | 0.23 | 0.33 | 0.24 | 0.42 | 0.33 |
| 1.8 | 0.31 | 0.22 | 0.31 | 0.22 | 0.35 | 0.24 | 0.40 | 0.32 |
| 2.1 | 0.26 | 0.20 | 0.29 | 0.19 | 0.31 | 0.22 | 0.39 | 0.28 |
| 2.5 | 0.24 | 0.13 | 0.24 | 0.15 | 0.26 | 0.15 | 0.33 | 0.24 |
| 3.1 | 0.19 | 0.11 | 0.20 | 0.11 | 0.22 | 0.12 | 0.29 | 0.20 |
| 4.5 | 0.16 | 0.06 | 0.17 | 0.08 | 0.17 | 0.08 | 0.21 | 0.12 |
| 6 | 0.17 | 0.07 | 0.15 | 0.07 | 0.18 | 0.09 | 0.23 | 0.15 |
| 8 | 0.15 | 0.07 | 0.10 | 0.03 | 0.17 | 0.06 | 0.18 | 0.08 |
| 10 | 0.15 | 0.05 | 0.13 | 0.04 | 0.12 | 0.03 | 0.14 | 0.05 |
| 20 | 0.10 | 0.01 | 0.11 | 0.00 | 0.13 | 0.03 | 0.14 | 0.03 |

| Noise Level | Lopsided L shaped | | Spike | | Cubic, Y stretched | | LP, medium frequency | |
| --- | --- | --- | --- | --- | --- | --- | --- | --- |
|  | ApproxMaxMI | ChiMIC | ApproxMaxMI | ChiMIC | ApproxMaxMI | ChiMIC | ApproxMaxMI | ChiMIC |
| 0 | 1 | 1 | 1 | 1 | 1 | 1 | 1 | 1 |
| 0.05 | 0.97 | 0.97 | 0.96 | 0.96 | 0.97 | 0.97 | 0.98 | 0.97 |
| 0.1 | 0.94 | 0.94 | 0.95 | 0.95 | 0.94 | 0.92 | 0.92 | 0.91 |
| 0.2 | 0.93 | 0.91 | 0.93 | 0.92 | 0.90 | 0.86 | 0.87 | 0.83 |
| 0.3 | 0.91 | 0.87 | 0.86 | 0.84 | 0.82 | 0.75 | 0.76 | 0.73 |
| 0.4 | 0.85 | 0.80 | 0.85 | 0.80 | 0.75 | 0.70 | 0.65 | 0.60 |
| 0.5 | 0.82 | 0.75 | 0.82 | 0.75 | 0.66 | 0.58 | 0.56 | 0.51 |
| 0.6 | 0.72 | 0.62 | 0.70 | 0.63 | 0.57 | 0.51 | 0.50 | 0.40 |
| 0.7 | 0.66 | 0.53 | 0.61 | 0.51 | 0.50 | 0.42 | 0.37 | 0.28 |
| 0.8 | 0.60 | 0.48 | 0.57 | 0.47 | 0.45 | 0.38 | 0.35 | 0.28 |
| 0.9 | 0.51 | 0.43 | 0.50 | 0.35 | 0.36 | 0.29 | 0.31 | 0.24 |
| 1 | 0.46 | 0.36 | 0.41 | 0.32 | 0.31 | 0.20 | 0.25 | 0.04 |
| 1.15 | 0.39 | 0.30 | 0.36 | 0.27 | 0.24 | 0.16 | 0.20 | 0.14 |
| 1.3 | 0.33 | 0.23 | 0.28 | 0.19 | 0.20 | 0.11 | 0.18 | 0.10 |
| 1.45 | 0.30 | 0.21 | 0.27 | 0.17 | 0.19 | 0.11 | 0.18 | 0.08 |
| 1.6 | 0.31 | 0.22 | 0.21 | 0.14 | 0.19 | 0.08 | 0.18 | 0.08 |
| 1.8 | 0.25 | 0.15 | 0.20 | 0.12 | 0.15 | 0.07 | 0.14 | 0.03 |
| 2.1 | 0.22 | 0.14 | 0.19 | 0.10 | 0.16 | 0.03 | 0.15 | 0.04 |
| 2.5 | 0.21 | 0.12 | 0.15 | 0.03 | 0.14 | 0.05 | 0.14 | 0.01 |
| 3.1 | 0.18 | 0.09 | 0.17 | 0.07 | 0.13 | 0.03 | 0.10 | 0.03 |
| 4.5 | 0.16 | 0.06 | 0.13 | 0.05 | 0.10 | 0.00 | 0.11 | 0.02 |
| 6 | 0.15 | 0.06 | 0.14 | 0.03 | 0.10 | 0.00 | 0.11 | 0.01 |
| 8 | 0.14 | 0.04 | 0.11 | 0.02 | 0.10 | 0.01 | 0.09 | 0.00 |
| 10 | 0.12 | 0.02 | 0.13 | 0.03 | 0.10 | 0.00 | 0.09 | 0.00 |
| 20 | 0.09 | 0.00 | 0.10 | 0.01 | 0.10 | 0.00 | 0.09 | 0.00 |

| Noise Level | Cubic | | Sin, low frequency | | VF [med] cos | | VF [med] sin | | Sin, high frequency | |
| --- | --- | --- | --- | --- | --- | --- | --- | --- | --- | --- |
|  | ApproxMaxMI | ChiMIC | ApproxMaxMI | ChiMIC | ApproxMaxMI | ChiMIC | ApproxMaxMI | ChiMIC | ApproxMaxMI | ChiMIC |
| 0 | 1 | 1 | 1 | 1 | 1 | 1 | 1 | 1 | 1 | 1 |
| 0.05 | 0.97 | 0.97 | 0.98 | 0.98 | 0.99 | 0.99 | 0.99 | 0.99 | 0.99 | 0.99 |
| 0.1 | 0.94 | 0.92 | 0.95 | 0.94 | 0.96 | 0.96 | 0.97 | 0.96 | 0.99 | 0.98 |
| 0.2 | 0.86 | 0.83 | 0.90 | 0.88 | 0.93 | 0.91 | 0.94 | 0.92 | 0.94 | 0.92 |
| 0.3 | 0.83 | 0.78 | 0.83 | 0.78 | 0.86 | 0.82 | 0.85 | 0.82 | 0.81 | 0.79 |
| 0.4 | 0.75 | 0.69 | 0.80 | 0.74 | 0.79 | 0.76 | 0.83 | 0.80 | 0.79 | 0.76 |
| 0.5 | 0.65 | 0.59 | 0.73 | 0.69 | 0.75 | 0.71 | 0.75 | 0.72 | 0.74 | 0.73 |
| 0.6 | 0.61 | 0.53 | 0.68 | 0.62 | 0.61 | 0.58 | 0.66 | 0.64 | 0.64 | 0.62 |
| 0.7 | 0.52 | 0.45 | 0.59 | 0.54 | 0.57 | 0.50 | 0.52 | 0.49 | 0.55 | 0.54 |
| 0.8 | 0.43 | 0.35 | 0.53 | 0.47 | 0.51 | 0.46 | 0.48 | 0.44 | 0.51 | 0.47 |
| 0.9 | 0.36 | 0.26 | 0.44 | 0.34 | 0.39 | 0.33 | 0.45 | 0.39 | 0.43 | 0.39 |
| 1 | 0.33 | 0.26 | 0.39 | 0.31 | 0.41 | 0.34 | 0.35 | 0.27 | 0.37 | 0.34 |
| 1.15 | 0.21 | 0.11 | 0.31 | 0.22 | 0.29 | 0.21 | 0.30 | 0.24 | 0.29 | 0.08 |
| 1.3 | 0.20 | 0.13 | 0.26 | 0.18 | 0.23 | 0.15 | 0.22 | 0.03 | 0.23 | 0.04 |
| 1.45 | 0.19 | 0.09 | 0.25 | 0.15 | 0.23 | 0.14 | 0.21 | 0.08 | 0.22 | 0.01 |
| 1.6 | 0.15 | 0.06 | 0.18 | 0.11 | 0.21 | 0.12 | 0.20 | 0.13 | 0.21 | 0.06 |
| 1.8 | 0.16 | 0.05 | 0.17 | 0.07 | 0.18 | 0.11 | 0.19 | 0.01 | 0.17 | 0.04 |
| 2.1 | 0.15 | 0.05 | 0.17 | 0.07 | 0.13 | 0.00 | 0.14 | 0.04 | 0.15 | 0.03 |
| 2.5 | 0.14 | 0.04 | 0.12 | 0.04 | 0.18 | 0.02 | 0.14 | 0.01 | 0.13 | 0.02 |
| 3.1 | 0.12 | 0.01 | 0.14 | 0.03 | 0.15 | 0.01 | 0.15 | 0.01 | 0.16 | 0.02 |
| 4.5 | 0.12 | 0.02 | 0.12 | 0.02 | 0.10 | 0.01 | 0.12 | 0.00 | 0.12 | 0.01 |
| 6 | 0.10 | 0.01 | 0.09 | 0.01 | 0.13 | 0.00 | 0.11 | 0.02 | 0.09 | 0.00 |
| 8 | 0.11 | 0.01 | 0.10 | 0.00 | 0.10 | 0.01 | 0.11 | 0.01 | 0.10 | 0.00 |
| 10 | 0.11 | 0.01 | 0.13 | 0.01 | 0.09 | 0.00 | 0.08 | 0.01 | 0.11 | 0.00 |
| 20 | 0.11 | 0.01 | 0.11 | 0.01 | 0.11 | 0.00 | 0.11 | 0.01 | 0.11 | 0.01 |

| Noise Level | NFF [low] cos | | Cos, high frequency | | Parabola | | NFF [low] sin | |
| --- | --- | --- | --- | --- | --- | --- | --- | --- |
|  | ApproxMaxMI | ChiMIC | ApproxMaxMI | ChiMIC | ApproxMaxMI | ChiMIC | ApproxMaxMI | ChiMIC |
| 0 | 1 | 1 | 1 | 1 | 1 | 1 | 1 | 1 |
| 0.05 | 0.99 | 0.99 | 1.00 | 1.00 | 0.98 | 0.98 | 0.99 | 0.99 |
| 0.1 | 0.95 | 0.95 | 0.96 | 0.95 | 0.95 | 0.94 | 0.97 | 0.96 |
| 0.2 | 0.91 | 0.87 | 0.90 | 0.88 | 0.89 | 0.86 | 0.91 | 0.88 |
| 0.3 | 0.86 | 0.81 | 0.85 | 0.84 | 0.85 | 0.80 | 0.87 | 0.84 |
| 0.4 | 0.78 | 0.73 | 0.78 | 0.75 | 0.82 | 0.75 | 0.82 | 0.77 |
| 0.5 | 0.73 | 0.68 | 0.69 | 0.68 | 0.78 | 0.70 | 0.72 | 0.67 |
| 0.6 | 0.63 | 0.57 | 0.64 | 0.62 | 0.68 | 0.60 | 0.63 | 0.60 |
| 0.7 | 0.59 | 0.52 | 0.57 | 0.55 | 0.63 | 0.55 | 0.59 | 0.56 |
| 0.8 | 0.52 | 0.45 | 0.48 | 0.42 | 0.58 | 0.51 | 0.54 | 0.47 |
| 0.9 | 0.42 | 0.37 | 0.41 | 0.36 | 0.49 | 0.42 | 0.42 | 0.36 |
| 1 | 0.37 | 0.30 | 0.38 | 0.35 | 0.45 | 0.37 | 0.37 | 0.31 |
| 1.15 | 0.35 | 0.26 | 0.29 | 0.12 | 0.30 | 0.22 | 0.27 | 0.21 |
| 1.3 | 0.25 | 0.19 | 0.29 | 0.22 | 0.28 | 0.18 | 0.26 | 0.21 |
| 1.45 | 0.25 | 0.17 | 0.21 | 0.14 | 0.25 | 0.15 | 0.22 | 0.17 |
| 1.6 | 0.21 | 0.14 | 0.23 | 0.08 | 0.21 | 0.12 | 0.20 | 0.12 |
| 1.8 | 0.19 | 0.10 | 0.20 | 0.02 | 0.18 | 0.09 | 0.17 | 0.08 |
| 2.1 | 0.16 | 0.07 | 0.14 | 0.01 | 0.16 | 0.07 | 0.19 | 0.03 |
| 2.5 | 0.14 | 0.02 | 0.18 | 0.00 | 0.18 | 0.08 | 0.13 | 0.05 |
| 3.1 | 0.12 | 0.05 | 0.11 | 0.02 | 0.14 | 0.04 | 0.12 | 0.02 |
| 4.5 | 0.10 | 0.00 | 0.10 | 0.02 | 0.11 | 0.03 | 0.12 | 0.01 |
| 6 | 0.12 | 0.01 | 0.12 | 0.01 | 0.11 | 0.02 | 0.10 | 0.01 |
| 8 | 0.12 | 0.01 | 0.09 | 0.02 | 0.10 | 0.02 | 0.12 | 0.01 |
| 10 | 0.13 | 0.01 | 0.11 | 0.01 | 0.11 | 0.00 | 0.10 | 0.02 |
| 20 | 0.11 | 0.01 | 0.11 | 0.01 | 0.10 | 0.00 | 0.10 | 0.00 |
